# Atypical Cutaneous Leishmaniasis Variant

**DOI:** 10.7759/cureus.19252

**Published:** 2021-11-04

**Authors:** Jaime A Hidalgo-Enríquez, Alberto Moscona-Nissan, Sayonara Zaputt-Cabrera, Laura I Rincón-Ángel, Anthony D Hidalgo-Enríquez

**Affiliations:** 1 School of Medicine, Universidad Espíritu Santo, Guayaquil, ECU; 2 School of Medicine, Universidad Panamericana, Mexico City, MEX; 3 Dermatology, Hospital José Carrasco Arteaga, Cuenca, ECU

**Keywords:** amastigotes, granuloma, infectology, verrucous plaque, cutaneous leishmaniasis

## Abstract

Leishmaniasis is a complex group of parasitic infectious diseases caused by intracellular protozoa of the genus *Leishmania*. It is a zoonosis mainly transmitted by the bite of infected female *Phlebotomus *or *Lutzomyia *sandflies. Clinical manifestations of leishmaniasis are diverse and can range from asymptomatic presentations to disseminated systemic disease. Cutaneous leishmaniasis is endemic in more than 80 countries in the world, having a predominance in tropical and subtropical regions. Although the majority of cases follow a classic development, an increasing number of new and rare variants of cutaneous leishmaniasis have been reported. These variants should be suspected as a cause of diverse clinical presentations, especially in endemic regions and travelers, being a diagnostic challenge for physicians.

We present a case of atypical cutaneous leishmaniasis found as a single verrucous plaque of eight months of evolution in the left posterior thigh of a 35-year-old man, who presented mild pruritus. The patient reported shrimp farming as his main occupational activity and was living in a rural region surrounded by forest on the Pacific coast of Ecuador. On dermatological examination, a single 4 x 5 cm verrucous plaque with irregular borders and a scaly erythematous violaceous aspect was found. Histopathological analysis revealed the presence of lymphohistiocytic inflammatory infiltrate with plasmocytes and granulomatous inflammation. On the Giemsa stain, intracellular amastigotes (Leishman-Donovan bodies) were observed. The treatment consisted of intramuscular meglumine antimoniate, presenting significant improvement on follow-up.

## Introduction

Leishmaniasis is a complex group of parasitic infectious diseases caused by intracellular protozoa of the genus *Leishmania*. It is a zoonosis mainly transmitted by the bite of infected female *Phlebotomus* or *Lutzomyia* sandflies [1,2]. Clinical manifestations of leishmaniasis are diverse and can range from asymptomatic presentations to disseminated systemic disease [1,2]. *Leishmania* species vary according to geographic areas and countries in the world and determine clinical features of these diseases depending on the location of the lesions: cutaneous, mucocutaneous, and visceral leishmaniasis (kala-azar).

Cutaneous leishmaniasis (CL) is endemic in more than 80 countries in the world, having a predominance in tropical and subtropical regions of South America and Asia [3]. It is characterized by the initial appearance of an erythematous papule in the site where an infected sandfly has bitten the patient. Afterward, patients present pruritus and erythema, and the lesion increases in size and depth, becoming an ulcer. CL ulcers are often covered by scabs and present serous exudate. CL lesions usually present in exposed parts of the body such as the face, arms, and legs. It can be associated with permanent scar formation, decreased quality of life, and psychological consequences, such as anxiety and depression [1-3].

Although the majority of cases (87-98%) follow a classic development and are considered classic cutaneous leishmaniasis (CCL), an increasing number of new and rare variants of CL have been reported, being called atypical cutaneous leishmaniasis (ACL). CL can be found as multiple clinical entities that represent a diagnostic challenge for physicians, who should consider these variants when assessing CL cases in endemic countries or in travelers. Many ACL variants have been described, such as erysipeloid, verrucous, dry, lupoid, zosteriform, paronychial, eczematous, sporotrichoid, psoriasiform, annular, necrotic, erythematous volcanic ulcer, vegetant, and chancriform presentations [3].

## Case presentation

A 35-year-old man presented to a rural health care center on the Pacific coast of Ecuador due to a slow-growing cutaneous lesion of eight months of evolution in the left posterior thigh, accompanied by mild pruritus, with no other complaints associated. The patient reported having presented a similar lesion seven years before, which resolved without treatment. Ten years prior to the evaluation, the patient was diagnosed with ulcerative colitis, which was managed with azathioprine during the past three years. The patient's main occupational activity was shrimp farming and he lived in a rural region surrounded by forest.

**Figure 1:**
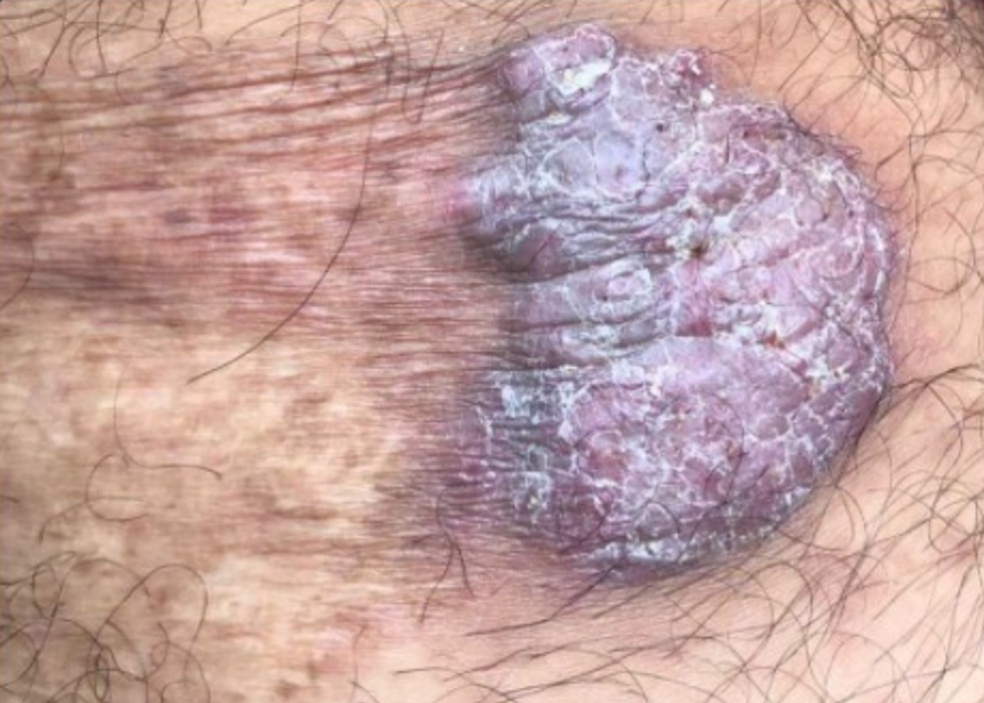
Verrucous plaque found on the left posterior thigh.

General physical examination of the patient was unremarkable and his vital signs were found within normal limits. On dermatological examination, a single 4 x 5 cm verrucous plaque with irregular borders and a scaly erythematous violaceous aspect was found on the patient's left posterior thigh (Figure 1).

**Figure 2:**
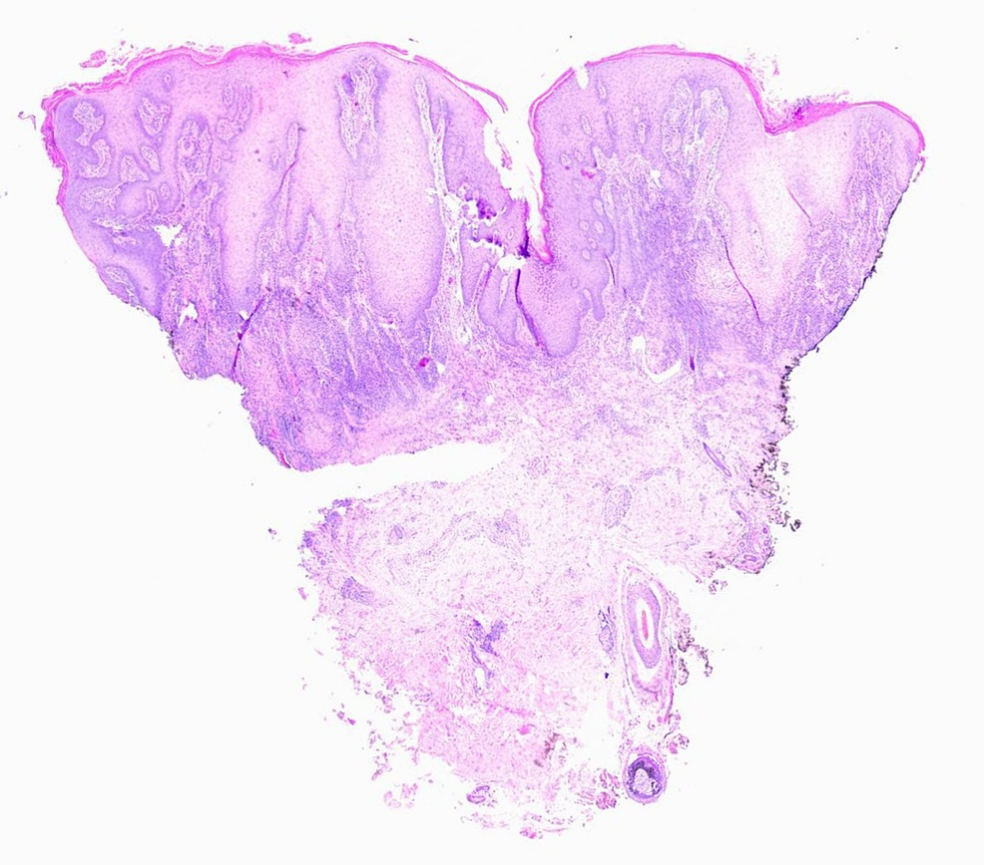
H&E stained histological section showing pseudoepitheliomatous hyperplasia and mixed inflammatory infiltrate in the papillary and reticular dermis. H&E: hematoxylin and eosin.

**Figure 3:**
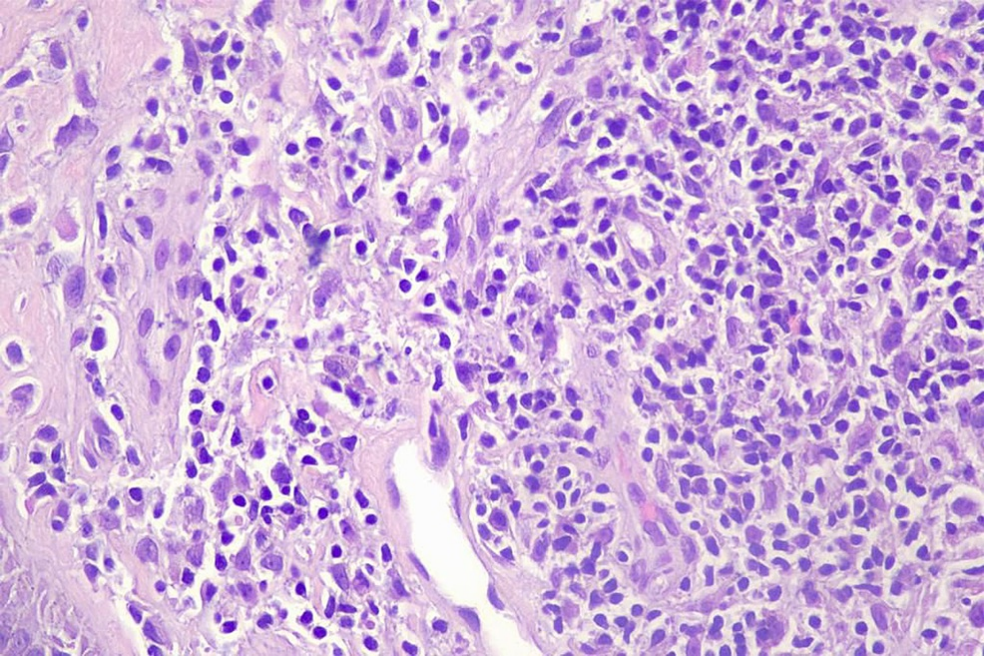
H&E stained histological section of dermis showing lymphohistiocytic inflammatory infiltrate with plasmocytes and intracellular granular material in histiocytes suggestive of a parasitic infection. H&E: hematoxylin and eosin.

**Figure 4:**
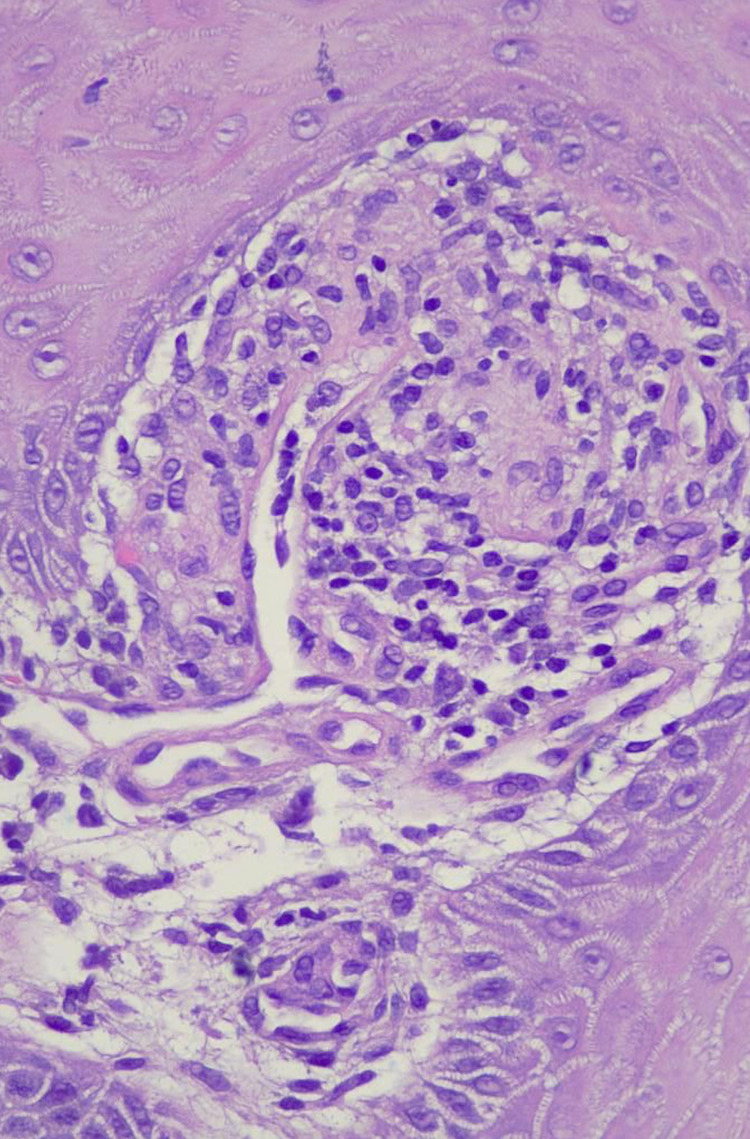
H&E stained histological section (40x) of papillary dermis showing the formation of a tuberculoid granuloma. H&E: hematoxylin and eosin.

**Figure 5:**
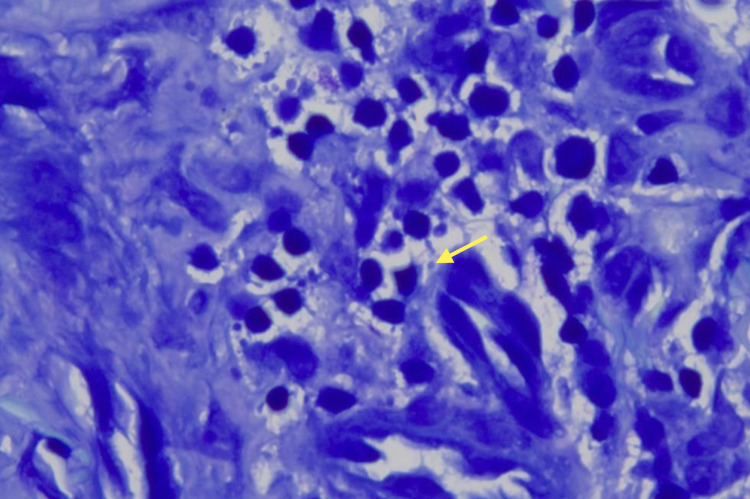
Giemsa stained histological section (100x) of dermis showing intracellular amastigotes (Leishman-Donovan bodies) in histiocytes.

The lesion had a firm consistency and was painless on palpation. Histopathological analysis revealed the presence of granulomatous inflammation, abundant multinucleated giant cells, and intracellular amastigotes on Giemsa stain (Figures 2-5).

Further laboratory studies such as complete blood count (CBC), liver function tests (LFTs), and metabolic panel were performed and found with no relevant alterations. A fourth-generation human immunodeficiency virus test (enzyme-linked immunosorbent assay) was done revealing negative results. Management of the patient consisted of intramuscular meglumine antimoniate of 20 mg/Kg. On the patient's follow-up after four weeks, significant improvement was observed.

## Discussion

Cutaneous leishmaniasis is endemic in more than 80 countries in the world, having a predominance in tropical and subtropical regions [2]. It is estimated that 1-1.5 million cases occur annually, affecting people living in endemic countries and travelers [2]. Most of the cases of cutaneous leishmaniasis are observed mainly in Algeria, Peru, Saudi Arabia, Syria, and Pakistan [1,4]. The geographical distribution of leishmaniasis also seems to be influenced by the type of *Leishmania* that caused the disease: *L. braziliensis* is found from Mexico to Argentina; *L. peruana* is reported in the Peruvian Andes; *L. guayaneses* is observed in Brazil, Suriname, Venezuela, Ecuador, Colombia, and French Guiana; *L. panamensis* is found in Colombia, Venezuela, Ecuador, Panama, and Costa Rica; *L. amazonensis* is found in the Amazon in Brazil; *L. mexicana* is found from the areas of Texas and Mexico to Colombia and Venezuela; and *L. venezuelensis* is found in Venezuela. The subtropical area in the northeast part of Ecuador is an endemic zone for cutaneous leishmaniasis [1,4].

Some risk factors for transmission are migration, war, the introduction of non-immune people to an endemic area, poor drainage and water management, overcrowding, and deforestation. These risk factors promote vector proliferation and access to human hosts; therefore, a more active transmission cycle. Male gender, adult age, working in rural areas, and socioeconomic conditions have also been found to be risk factors for contracting the disease [4,5].

*Leishmania* is a flagellated protozoan from *Zoomastigophora* class, *Kinetoplastida* order, and *Trypanosomatidae* family. *Leishmania* includes more than two dozen species, most of which are capable of parasitizing humans. They appear in an intracellular form, amastigote, within the reticuloendothelial system. Amastigotes are presented as Leishman-Donovan bodies in histopathology. This microorganism usually has visceral tropism or mucocutaneous tropism (depending on the species) [5].

The vectors of cutaneous leishmaniasis are *Diptera,* *Nematocera, Phlebotomus,* and *Lutzomyia*. They are present in intertropical zones and temperate zones. The disease transmission cycle consists of the female sandfly becoming infected by ingesting blood from the infected mammal. In the digestive tract of the vector, the parasites change from amastigote to promastigote. Promastigote then changes from the nectomonal phase to haptomone phase and then to metacyclic promastigote. Metacyclic promastigotes remain in the pharynx until inoculated into a new host. Parasites are phagocytosed by macrophages in the new host and become amastigotes once again [1,5].

The different forms of clinical presentation of this parasitic infection are the result of a complex interaction between some factors like the response of the immune system and genetics of the affected patient, the species of the parasite, and vector-dependent factors [6]. Infection with *Leishmania spp*. can manifest as asymptomatic and be unnoticed by the patient; on the other hand, it can be expressed as one of the four different forms of the disease. The classic form consists in the appearance of an erythematous zone at the site of the bite of the vector on the host and, subsequently, a papule grows, then progresses into a nodule, and finally in a variable period of time, develops into an ulcer, which is the typical lesion of what is known as localized cutaneous leishmaniasis (LCL) [4,6]. LCL is the most common clinical form (61-98% of cases) and may achieve spontaneous resolution without intervention [6]. However, skin lesions can spread to other sites, which is known as disseminated cutaneous leishmaniasis (DCL), which has been typically linked to immunosuppression [6,7]. An LCL variant that has also been described in the literature is *Leishmaniasis recidivans*, which is characterized by achieving spontaneous resolution and years later presenting a new lesion adjacent to the healed scar [3,4].

It may even be the case of greater expansion when affecting mucous membranes mainly of the nose, mouth, and larynx, a clinical presentation known as mucocutaneous leishmaniasis (MCL) [7]. The parasite is also able to spread and generate damage into organs such as the liver, spleen, and bone marrow as occurs in visceral leishmaniasis (VL), also known as kala-azar. VL is the most severe clinical form of this parasitosis in which the patient has irregular episodes of fever accompanied by anemia, hepatosplenomegaly, and weight loss [7].

Considering that the most common clinical presentation of the disease is CCL, it is also important to mention that a patient may develop an atypical lesion (ACL), which is one of the main contributors to delays in diagnosis and treatment. Due to the atypical forms of LCL, this infectious disease has been recognized as “the great imitator” [8]. Some of the presentations that have been reported are tumor or squamous cell carcinoma-like, erysipeloid, eczematous, psoriasiform, verrucous, dry, zosteriform, paronychial, sporotrichoid, annular, necrotic, discoid lupus erythematosus like, and acneiform, among others [3,8].

We presented a case of atypical cutaneous leishmaniasis found as a single verrucous plaque of eight months of evolution in the left posterior thigh of a 35-year-old man who was immunocompromised due to azathioprine treatment for ulcerative colitis. Additionally, the patient reported having presented a similar lesion seven years before, which resolved without treatment. To initiate early management and improve patient prognosis, ACL variants should be considered as a cause of diverse clinical presentations, especially in leishmaniasis endemic regions and travelers. It is fundamental for physicians to suspect ACL, given epidemiologic factors of the patient, comorbidities, and distinct clinical presentations of this disease, being a diagnostic challenge and differential diagnosis for multiple clinical entities.

CL histopathologic findings include granulomatous inflammation with abundant multinucleated giant cells and observation of parasites (amastigotes) with Giemsa or Wright stain [9]. A skin biopsy reveals the presence of vacuolated histiocytes and Leishman-Donovan bodies or amastigotes [9]. Additionally, leishmaniasis may be diagnosed through a Montenegro test (MT), which is an immunological delayed hypersensitivity reaction. MT has high sensitivity and specificity but it does not allow to establish differences in current or past infection. It consists of the intradermal application of 0.1 mm of deactivated promastigotes on the anterior face of the forearm, and 48 to 72 hours later, direct visualization is performed [10,11]. MT is considered as positive if an induration greater than 5 millimeters develops on the intradermal application site and negative with measurement less than 5 millimeters. It is estimated that leishmaniasis endemic populations usually test positive by this method, so direct visualization of parasites on a smear, biopsy, or molecular techniques as polymerase chain reaction is preferred for diagnosis [10,11].

Cutaneous leishmaniasis can be treated with systemic treatment (indicated in lesions larger than 4 cm, multiple lesions, affected joints, hands, feet, face, and in immunosuppressed patients) or with local treatment. The treatment administration can be either topical, intralesional, subcutaneous, intramuscular, oral, or intravenous [12].

Topical treatment usually consists of paromomycin with methylbenzethonium chloride, paromomycin with gentamicin, paromomycin alone (paromomycin alone has been used primarily for *L. major*), and even topical amphotericin B. Intralesional treatment is usually used with antimony drugs [13]. The benefit of intralesional treatment compared to systemic therapy is lower toxicity. Sodium stibogluconate can be used in this treatment modality. In subcutaneous therapy, only formulations with oral miltefosine have significant therapeutic effects. Pentamide and meglumine antimoniate can be used for intramuscular treatment. As mentioned above, miltefosine, ketoconazole, allopurinol, and fluconazole can be used for oral treatment [13]. Amphotericin B, miltefosine, pentamide, and pentavalent antimony can be used for intravenous treatment. Liposomal amphotericin is a treatment of choice throughout the world. The effectiveness of treatment depends on the use of a specific drug for each type of *Leishmania* and its sensitivity to the drug. Physical treatment is also used in many modalities such as cryotherapy, thermotherapy, surgery, curettage, ulcer debridement, CO2 laser, and electrodesiccation. Prevention should be the priority goal for the control of leishmaniasis. Vaccination, reducing the exposure to flies, and government measures and sanitary policies are some of the main steps for this disease prevention [13].

## Conclusions

Although the majority of cases of cutaneous leishmaniasis (87-98%) follow a classic development, skin ulcers being the most characteristic feature of this disease, an increasing number of CL variants have been reported. Atypical cutaneous leishmaniasis can be found as multiple clinical entities that represent a diagnostic challenge for physicians, who should consider these variants when assessing CL cases in endemic countries or in travelers. Some of CL differential diagnoses are fungal and bacterial skin infections and neoplasms. To initiate early management and improve patient prognosis, physicians should consider these variants as a differential diagnosis of multiple clinical entities.
